# The Lateral Movements in the Knee-Joint in Cases of White Swelling

**Published:** 1877-05

**Authors:** 


The Lateral Movements in the Knee-Joint in Cases of White Swelling.—These movements have been ascribed by surgical writers exclusively to alterations in, or destruction of, the peripheral ligaments. M. Moutard-Martin, however, has had opportunities of observing four cases which presented this sign during life, and in which the autopsies showed that the ligaments retained their normal strength, while the cartilages were greatly altered, or even absolutely destroyed. The first case was one of suppurative arthritis, consecutive to osteo-myelitis, and the lateral movements during life were attended by crepitation. The autopsy showed destruction of the cartilages, but the anterior, posterior, and lateral ligaments were healthy; only the accessory fibrous bands which form the capsule presented fungous growths. The three other cases were instances of white swelling, and in none of them was crepitation present during life. In two of these cases the internal surfaces of the capsular ligaments were covered with fungous growths, and in one there was an opening through the internal ligament into the bursa that separated the gracilis from the knee-joint. In all three cases, however, the true ligaments preserved their normal thickness and strength; they were neither relaxed nor softened. On the other hand, the cartilages were either thinned and atrophied, or were softened, ulcerated, and destroyed in part, so that the space between the bones was incompletely filled out. In the first case, both cartilages and fibro-cartilages had entirely disappeared. These changes in the cartilages are quite sufficient to explain the abnormal lateral mobility. In one of the cases the crucial ligaments were still intact. It is quite possible that in all these cases, if life had been prolonged, the abnormal movements would ultimately have produced stretching and disorganization of the true ligaments. If these observations are confirmed by further autopsies, lateral mobility will become an important indication for the production of anchylosis, or for amputation or resection.—*Le Progres Medical*, Feb. 3, 1877.

